# *Ex vivo* analysis platforms for monitoring amyloid precursor protein cleavage

**DOI:** 10.3389/fnmol.2022.1068990

**Published:** 2023-01-06

**Authors:** Yuji Kamikubo, Hao Jin, Yiyao Zhou, Kazue Niisato, Yoshie Hashimoto, Nobumasa Takasugi, Takashi Sakurai

**Affiliations:** Department of Pharmacology, Juntendo University School of Medicine, Tokyo, Japan

**Keywords:** Alzheimer’s disease, amyloid β, neurodegenerative disease, organotypic brain culture, hippocampus, secretase

## Abstract

Alzheimer’s disease (AD) is a progressive neurodegenerative brain disorder and the most common cause of dementia in the elderly. The presence of large numbers of senile plaques, neurofibrillary tangles, and cerebral atrophy is the characteristic feature of AD. Amyloid β peptide (Aβ), derived from the amyloid precursor protein (APP), is the main component of senile plaques. AD has been extensively studied using methods involving cell lines, primary cultures of neural cells, and animal models; however, discrepancies have been observed between these methods. Dissociated cultures lose the brain’s tissue architecture, including neural circuits, glial cells, and extracellular matrix. Experiments with animal models are lengthy and require laborious monitoring of multiple parameters. Therefore, it is necessary to combine these experimental models to understand the pathology of AD. An experimental platform amenable to continuous observation and experimental manipulation is required to analyze long-term neuronal development, plasticity, and progressive neurodegenerative diseases. In the current study, we provide a practical method to slice and cultivate rodent hippocampus to investigate the cleavage of APP and secretion of Aβ in an *ex vivo* model. Furthermore, we provide basic information on Aβ secretion using slice cultures. Using our optimized method, dozens to hundreds of long-term stable slice cultures can be coordinated simultaneously. Our findings are valuable for analyses of AD mouse models and senile plaque formation culture models.

## Introduction

Organotypic brain cultures, including slice cultures, are used to study the central nervous system owing to their advantages over both *in vivo* and *in vitro* research platforms (Cho et al., 2007; Uesaka et al., 2018). Sophisticated cultured brain slices retain tissue structure, neural circuit, and extracellular environment and replicate the native status of the *in vivo* context. Hippocampal slice cultures are widely used to investigate the effects of pharmacological, biochemical, physiological, and genetic manipulation on neuronal and glial cells (Bergold et al., 1993; Ikegaya, 1999). Three typical methods are used to maintain neural tissue *in vitro*: roller-tube cultures, matrix-embedded cultures, and membrane interfaces (Gähwiler et al., 1997). The membrane interface brain slice culture was improved for practical use by Stoppini et al. (1991). Since a sterilized filter membrane cup is designed to easily change the medium, this apparatus as an interface is helpful for the long-term maintenance of the explant tissue. Cultured hippocampal slices on the filter membrane cup were amenable to complete replacements of the culture medium. These advantages allowed us to keep the brain slices in culture for several months. Previously, we reported on neural circuits, synaptic plasticity, neurodevelopment, and neurodegenerative diseases using cultured hippocampal slices (Tominaga-Yoshino et al., 2002; Shinoda et al., 2005; Kamikubo et al., 2017).

Alzheimer’s disease (AD) is a chronic neurodegenerative brain disorder that usually starts slowly and worsens over a long period and is the most common cause of dementia in the elderly. Numerous genetic and biochemical studies have supported the hypothesis that excessive accumulation of amyloid β peptide (Aβ) results in aggregation and amyloid deposition in the brains of patients with AD (amyloid hypothesis) (Hardy and Selkoe, 2002). Aβ is derived from the amyloid precursor protein (APP), an integral type I membrane protein expressed in many tissues and concentrated in synapses. APP is commonly cleaved by membrane proteases in the secretase family: α-, β-, and γ-secretase. Aβ is generated by sequential cleavage by β-secretase (BACE1) (Vassar et al., 1999) and γ-secretase, whereas alternative cleavage by α-secretase prevents Aβ production (De Strooper and Annaert, 2000). Since neuronal activity is known to be an important regulator of APP β cleavage (Kamenetz et al., 2003; Sakurai et al., 2008), the regulation of cleavage, accumulation, and elimination of Aβ have been explored as a target for the therapy and prevention of AD.

Aβ is the main component of highly insoluble senile plaques in AD brain, but it is also known to form soluble and highly toxic aggregate, oligomers. Although amyloid plaques have been the first focus of study in the amyloid hypothesis, it has recently become clear that many toxic factors, including Aβ oligomers (Cline et al., 2018), are present. Aβ and other peptide oligomers have been reported to cause cytotoxicity and neurodegeneration (Caruso et al., 2019). On the other hand, APP cleavage products other than Aβ have been reported to be neurotoxic or neuroprotective (Chasseigneaux and Allinquant, 2012; Willem et al., 2015; Kaneshiro et al., 2022). Furthermore, previous studies showed that Aβ itself induces the secretion of neuroprotective factors (Zimbone et al., 2018). To elucidate the early pathogenesis of AD, it is necessary to clarify the function and behavior of Aβ monomers, oligomers, and other APP cleavage products.

A large amount of data from cell line model-based assays has revealed the molecular mechanism of Aβ secretion to understand AD (Hardy and Selkoe, 2002). Although cell line models are powerful tools for molecular biological studies, discordance has been observed between cell lines and animal models. Since neuronal and glial cells are morphologically and functionally specialized, dissociated primary cultures of neuronal tissues have the advantage of revealing the pathological processes and molecular mechanisms of neuropsychiatric disorders. Although experiments using cultured neurons provide important information, they exhibit loss of the tissue architecture of the brain. Since neural circuits, glial networks, and extracellular environments are essential in neurodevelopment, and neurodegenerative diseases, including AD, analysis using cultured neurons alone is insufficient (Vassar et al., 1999; Ichikawa-Tomikawa et al., 2012; Irie et al., 2012; Sethi and Zaia, 2017). However, several disadvantages are associated with animal model experiments, including the requirement of a long duration, substantial cost, and laborious monitoring of multiple parameters following manipulations. Furthermore, genetically engineered animals may be affected by compensatory and developmental changes (Kreiner, 2015). For this reason, hippocampal slice culture may be a potential technique for linking animal models and neural cells.

In the current study, we describe the methods of organotypic hippocampal slice culture and consecutive analysis of Aβ and related products, which play a central role in the pathogenesis of AD. The hippocampus is thought to be one of the first brain regions to suffer damage in AD (Hyman et al., 1984). With the method reported here, we could produce nearly 100 slices in a single cultivation and maintain hippocampal slices for more than 1 month resulting in an increased analysis efficiency. Continuous collection and analysis of Aβ is a powerful approach for elucidating the cellular and molecular mechanisms underlying AD. Long-term and consecutive analyses are also required to develop and evaluate therapeutic agents and treatment approaches for AD.

## Materials and methods

### Reagents for slice culture

Slice culture medium (SCM) contained 50% minimal essential medium based on Earle’s salts (Nacalai Tesque, Kyoto, Japan, 21,442–25) with 72 mM glucose and 2 mM HEPES added, 25% Hank’s Balanced Salt Solution (Thermo Fisher Scientific, 24,020–117, MA, United States), and 25% horse serum that was heat-inactivated at 56°C for 30 min (Thermo Fisher Scientific, 16,050–122). Dissection solution was ice-cold Gey’s BSS (GBSS) (137 mM NaCl, 5 mM KCl, 0.18 mM KH_2_PO_4_, 0.84 mM Na_2_HPO_4_ 12H_2_O, 36 mM glucose, 1.5 mM CaCl_2_, 1 mM MgCl_2_, and 0.32 mM MgSO_4_) saturated with O_2_. Phosphate-buffered saline (PBS). 4% paraformaldehyde in Phosphate Buffer Solution (Nacalai Tesque, 09154–85). α-secretase inhibitor: GI 254023X (Tocris Bioscience, Bristol, United Kingdom, 3995), β-secretase inhibitor: BACE inhibitor IV (Calbiochem, CA, United States, 565788), γ-secretase inhibitors: N-(N-(3,5-difluorophenacetyl)-L-alanyl)-S-phenylglycine t-butyl ester (DAPT, Tokyo Chemical Industry, Tokyo, Japan, D4257). Primary antibodies used were anti-NeuN (a marker protein for neuronal nuclei) (mouse monoclonal, A60, Chemicon, CA, United States); anti-glial fibrillary acidic protein (GFAP) (a marker protein for astrocytes) (mouse monoclonal, SMI22, BioLegend, CA, USA); anti-ionized calcium-binding adapter molecule 1 (Iba1) (a marker protein for microglia) (rabbit, 019–19,741; Wako Pure Chemical Industries, Osaka, Japan); anti-BACE1 (rabbit monoclonal) (D10E5, Cell signaling Technology, MA, USA), and anti-APP (mouse monoclonal, 22C11, Millipore, Carrigtwohill, Ireland), anti-nicastrin (a membrane of γ-secretase complex) (mouse, 9C3, Biolegend) antibodies. The secondary antibodies used were goat anti-mouse IgG (Alexa Fluor 488, A-11001), goat anti-mouse IgG (Alexa Fluor 594, A-11032), goat anti-rabbit IgG (Alexa Fluor 488, A-11034), and goat anti-rabbit IgG (Alexa Fluor 594, A-11037) (Thermo Fisher Scientific, MA, USA). RIPA buffer consisted of 1% Nonidet P-40, 0.5% sodium deoxycholate, 0.1% SDS, 25 mM Tris–HCl, pH 7.5, 137 mM NaCl, and 3 mM KCl with a protease inhibitor cocktail (Complete, Roche Diagnostics, Mannheim, Germany). Furthermore, 1× Tris-buffered saline (TBS) consisted of 50 mM Tris, 138 mM NaCl, and 2.7 mM KCl. TBST consisted of 1× TBS with 0.1% Tween 20 added. The mouse/rat amyloid β(1–40) assay kit (27720), mouse/rat amyloid β(1–42) assay kit (27721), mouse/rat sAPPα (highly sensitive) assay kit (27419), and mouse sAPPβ-w Assay Kit (27416) were purchased from IBL, Gunma, Japan.

### Apparatus for slice culture

Tissue chopper (McIlwain), double-edge stainless steel cutting blades (FEATHER, FA-10), disposable scalpel (FEATHER, No.11, No. 22), filter membrane cup (Millicell-CM, Millipore, PICM03050 or PICM0RG50), stereomicroscope (Olympus, Tokyo, Japan, SZX7), and overhead projector (OHP) sheet cut into 5-cm squares were used for slice culture.

### Hippocampal slice culture method

All animal experiments were conducted in strict accordance with the institutional guidelines of Juntendo University for animal experiments. The protocol was approved by the Animal Experimentation Committee of Juntendo University (no. 845, 1,187). All animals were kept in an environment at 24°C with a 12-h light/dark cycle, with water and food freely available. At the end of the experiment, the animals were euthanized by exposure to carbon dioxide (CO_2_) or decapitation. Death was verified by respiratory arrest and dilated pupils. All surgical procedures were performed under carbon dioxide (CO_2_) anesthesia, and every effort was made to minimize animal suffering.

The hippocampal slice culture procedure in this study was an improvement of the method used in previous studies (Tominaga-Yoshino et al., 2002; Kamikubo et al., 2006, 2017). In this experiment, the Sprague–Dawley rat (postnatal days (P) 6–8, *n* = 80 in total) and Institute of Cancer Research (ICR) mice (P 7–8, *n* = 12 in total) purchased from Nihon SLC (Hamamatsu, Japan). For slice culture, adult rats or mice were euthanized by CO_2_, pups were euthanized by decapitation after CO_2_-anesthesia (Turner et al., 2020). The hippocampi were isolated after euthanasia.

**Figure 1:**
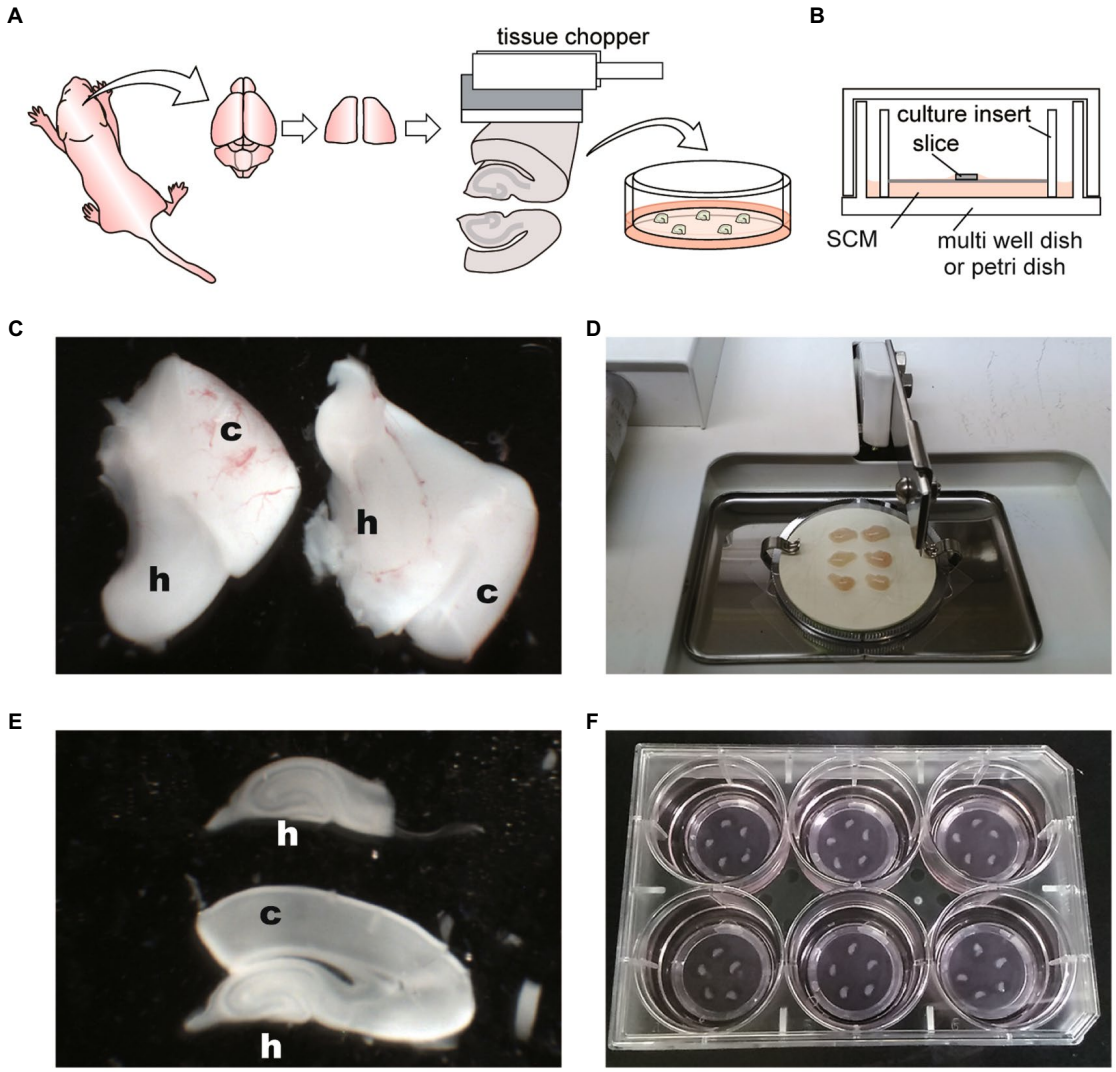
Schematic diagrams and photographs of procedure for hippocampal slice culture. **(A)** Schema of hippocampal slice culture method. **(B)** Schema representing disposition of slice on membrane culture insert and within the culture dish. SCM: slice culture medium. **(C)** Image of isolated hippocampi (H) with cerebral cortex (c). **(D)** Image of tissue chopper and isolated hippocampi with the cerebral cortex. Six hippocampi were placed on an OHP sheet in the cutting chamber. **(E)** Sliced hippocampus (h) with the cortex (c) and isolated hippocampus. **(F)** Five slices were placed onto the filter membrane cup in the six-well plate with SCM. Hippocampal slices are not located close to the cup walls of the inserts or close to each other.

The 400 μm thick hippocampal slices were obtained from the central region of the hippocampus using a tissue chopper with a cutting blade (Figure 1A). The slices were placed in the center of the filter membrane cups, and SCM was added to the bottom surface of the filter (Figure 1B). The prepared cultures were maintained at 37°C and 5% CO_2_. The culture medium was replaced twice per week with fresh medium throughout the culture period.

### Protocol

The optimized protocol developed based on previous studies on long-term culture of multiple hippocampal slices is as follows (Stoppini et al., 1991; Kamikubo et al., 2021). Since sterile slicing is critical for long-term culture, we modified previous slicing method to use sterile OHP sheets (Step 7–9). To avoid damaging the hippocampus, we modified previous method of dividing the hippocampal slices one by one (Step 10).

1. Wipe the stereomicroscope, tissue chopper, and micropipette with 70% ethanol solution. Sterilize the dissecting instruments and a cutting blade by immersing them in a 70% ethanol solution for 30 min. In addition, sterilize them for 30 min with UV light on a clean bench.
2. Dispense GBSS into a sterilized 100 ml bottle and place it on an ice box. Bubble the GBSS with 100% O_2_ for 20–30 min (40–50 ml GBSS is required per culture session [2–4 pups]). Owing to the high oxygen demand of the central nervous system, the working solution needs to be sufficiently oxygenated and cooled. Oxygen is blown through a long needle with a syringe filter to aseptically oxygenate the working solution.
3. Place the tissue chopper on a clean bench, attach a sterilized blade, and place the autoclaved OHP sheet on the cutting chamber.
4. Prepare a 6-well plate or 35-mm dishes, add 1 ml of SCM per well, and place a culture insert in each well. Carefully check that the filter membrane of the insert is completely wet and free of air bubbles underneath. Place the plates or dishes with culture inserts maintained at 37°C, 5% CO_2_, until needed.
5. The rodent pups were euthanized by decapitation after CO_2_-anesthesia. Cut the scalp along the midline to expose the skull. Cut along the sagittal suture and remove the skull from the rostral to the caudal. Quickly scoop out the brain with a micro medicine spoon and place it in oxygenated GBSS on ice.
6. Place the brain on an autoclave filter paper soaked in ice-cold oxygenated GBSS in a 100-mm dish. Using a scalpel, remove the midbrain and cerebellum, and then separate the hemisphere.
7. Under the stereomicroscope, gently remove the thalamus. The hippocampi are then exposed on each hemisphere in ice-cold oxygenated GBSS. Then, gently scoop the hippocampus out with the cortex using a micro spoon (Figure 1C).
8. Transfer the hippocampus to the OHP sheet on the tissue chopper chamber. Align the hippocampi perpendicular to the blade, drain excess GBSS, and obtain the coronal sections (Figure 1D).
9. Slice the hippocampi every 400 μm using a tissue chopper. Since tissue chopper and OHP sheet are placed at room temperature, quick operation is required in slicing the brain.
10. Transfer sliced hippocampi from the OHP sheet to a 60-mm dish filled with ice-cold oxygenated GBSS. Slicing with an OHP sheet underneath allows the slices to be easily separated in a sterile environment.
11. Under a stereomicroscope (Olympus, SZX7), pinch the cortex with precision tweezers, and divide the undamaged hippocampal slices one by one. Next, split it into the cerebral cortex and hippocampus with a scalpel (Figure 1E). It is essential to complete steps 5 to 10 within 45 min.
12. Incubate separated hippocampi in fresh oxygenated GBSS on ice for 30–60 min.
13. Transfer individual slices onto the culture inserts in the 6-well plate or 35-mm dish with pre-warmed SCM. Place 1–6 slices on one culture insert without placing slices near the walls of the inserts or close to each other (Figure 1F).
14. Return the plate or dish to the incubator, and maintain the slices at 37°C with a 5% CO_2_-enriched humidified atmosphere.
15. Replace with fresh SCM once every 3–4 days during the culture period. Aspirate the medium in a dish, and add 750–800 μl of pre-warmed fresh SCM per well. Approximately 200–250 μl of medium remains in the culture dish and filter membrane. When the sliced brain is immersed in liquid, oxygen deprivation damages nerve cells. To avoid neural cell death, the brain slice is exposed to air and medium that exudes from the filter membrane.

### Immunohistochemical staining

For the immunohistochemical staining, the cultured slices were fixed with 4% paraformaldehyde in PBS for 1 h at 4°C. The fixed preparations were rinsed four times with PBS and then treated with PBS containing 0.5% Triton X-100 and 5% fetal bovine or horse serum at 24°C for 30 min. The treated slices were incubated with a primary antibody against NeuN (1:200 dilution), GFAP (1:200), and Iba1 (1:100) at 4°C for 24 h. Slices treated with primary antibody and washed five times with PBS were incubated with a secondary antibody conjugated with Alexa Fluor 488 or 594 (1:400 dilution) at 24°C for 2–2.5 h (Kamikubo et al., 2006). Cultured slices were examined using an inverted microscope (Olympus, IX71) with an Orca-ER cooled CCD camera (Hamamatsu Photonics, Hamamatsu, Japan) or a Leica SP5/TCS confocal laser scanning microscope (Leica Microsystems, Wetzlar, Germany) (Sakairi et al., 2020).

### Propidium iodide staining

Propidium iodide (PI; Nacalai) is a red-fluorescent nuclear and chromosome counterstain. Since live cells are not permeable to PI, it is commonly used to detect dead cells in a population. We used PI fluorescence intensity as an index of total neuronal degeneration. Cultured slices were exposed to SCM containing 10 μg/ml PI for 5 h. Subsequently, slices were treated with 100 μM kainic acid (KA) for 3d to induce neuronal cell death. The whole slice was photographed with identical incident light intensity and exposure time on the fluorescence microscope (IX70 with a 4x objective lens, Olympus). PI fluorescence intensity was measured on all micrographs using MetaMorph software (Molecular Devices, CA, USA).

### Electrophoresis and immunoblotting

Acute or cultured hippocampal slices were solubilized in ice-cold RIPA buffer containing a protease inhibitor cocktail (Complete, Roche Diagnostics, Mannheim, Germany). The samples were resolved by SDS-PAGE and transferred onto polyvinylidene fluoride membranes (Immobilon-P, Millipore) (Kamikubo et al., 2017). Membranes were incubated in 5% skim milk in TBST at 25°C for 30–60 min to block nonspecific binding. Membranes were incubated overnight at 4°C or at 25°C for 2 h with primary antibodies against GFAP (BioLegend), Iba1 (Wako Pure Chemical Industries), anti-galectin-3 (mouse monoclonal, A3A12; Santa Cruz, CA, USA), or anti-β-actin (a loading control; mouse monoclonal; Wako Pure Chemical Industries). After four changes of 1× TBST and three 5-min washes at 25°C, the membranes were incubated in horseradish peroxidase-conjugated secondary antibodies. After four washes at 25°C, the membranes were incubated with an enhanced chemiluminescence solution (ECL western blotting substrate, Thermo Fisher Scientific, MA, USA). For quantification, chemiluminescence light signals in the Super Signal Dura substrate (Thermo Fisher Scientific) were captured using a cooled charge-coupled device camera system (LAS-3000plus; Fuji Photo Film Company, Kanagawa, Japan) that ensured a wide range of linearity. The densitometric quantification of protein expression was normalized to that of GAPDH or β-actin. Protein content was estimated using bicinchoninic acid (BCA, Thermo Fisher Scientific) (Kamikubo et al., 2017).

### Enzyme immunoassay for amyloid β and soluble APP

Aβ and soluble APP (sAPP) concentrations in the SCM were measured using an ELISA kit, as previously described (Kamikubo et al., 2017). The levels of Aβ_40_, Aβ_42_, sAPPα, and sAPPβ were measured using a solid-phase sandwich ELISA kit according to the supplier’s information (IBL, Gunma, Japan). To quantify Aβ production sequentially, we collected all the culture medium incubated with cultured slices and added 750 μl of the new culture medium. Aβ_40_ was quantified by diluting the medium by 10-to 25-fold, and Aβ_42_ was quantified by diluting the culture medium by 2-to 8-fold. Since Aβ quantification was performed in duplicate, we collected approximately 200 μl of the culture medium.

### Drug application

We assessed secretase dependency on APP processing in the culture system using α-, β-, and γ-secretase inhibitors (GI254023X, BACE inhibitor IV, and DAPT). Briefly, we incubated cultured hippocampal slices (14 DIV) or dissociated hippocampal cells (14 DIV) with 10 μM DAPT, secretase inhibitor mix (10 μM DAPT, 50 μM GI254023X, and 2 μM BACE inhibitor IV), or vehicle (DMSO) for 24 h. The GI254023X, BACE inhibitor IV, and DAPT were dissolved in DMSO (Sigma-Aldrich, MI, United States) to concentrations 500 and 1,000 times higher than the final levels, respectively, and kept at −20°C until use. GI 254023X and BACE inhibitor IV were added to fresh SCM before the analysis (Kamikubo et al., 2017). Other test drugs were dissolved in water to concentrations 1,000 times higher than the final levels, kept at 4 or − 20°C until use, and diluted into medium unless otherwise stated.

### Statistical analysis

All quantitative data are presented as mean ± standard deviation (SD). Statistical analysis was performed using either Student’s *t*-test (two-group comparison) or ANOVA (more than two groups), followed by a *post hoc* comparison. Multiple comparisons between groups were made by Tukey–Kramer test. The level of significance is indicated by asterisks: *p < 0.05, **p < 0.01, ***p < 0.001.

## Results

### Chronological analysis of cultured hippocampal slices

**Figure 2:**
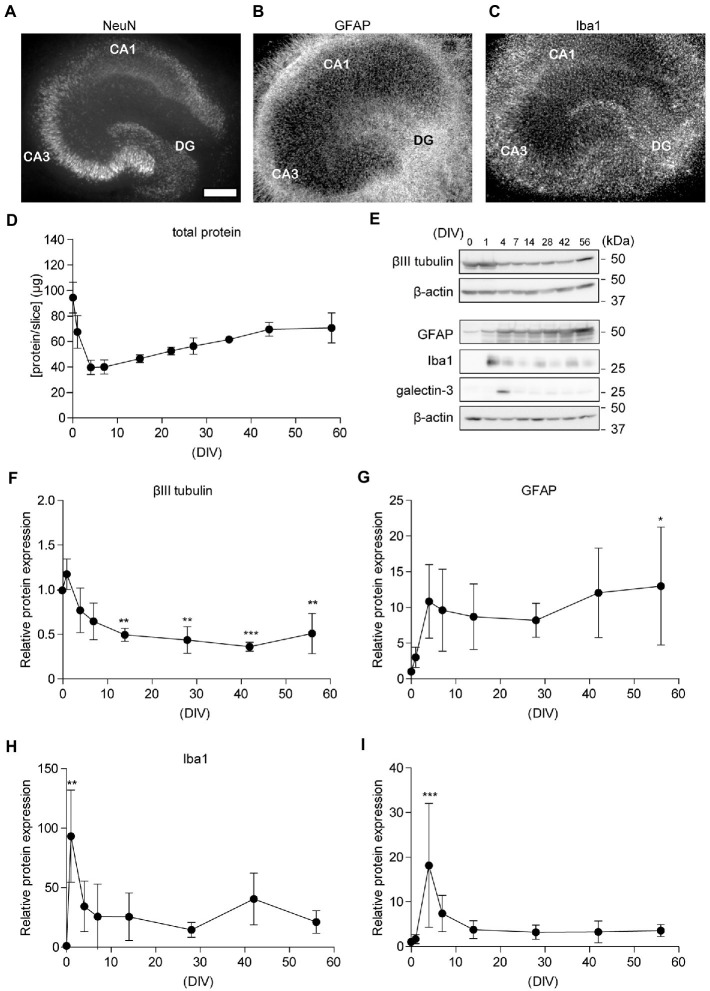
Chronological analysis of neuronal cells in cultured hippocampal slices. **(A–C)** Immunofluorescence staining of cultured hippocampal slices antibodies against NeuN **(A)**, GFAP **(B)**, and Iba1 **(C)**. CA1, Cornu Ammonis 1; CA3, Cornu Ammonis 3; DG, dentate gyrus. The scale indicates 500 μm. **(D)** Quantitative analysis of total protein in the lysate from four acute slices (0 days *in vitro*; DIV) or cultured slices (1–56 DIV). **(E–I)** Immunoblotting analysis of hippocampal slices with antibodies against βIII tubulin **(F)**, GFAP **(G)**, Iba1 **(H)**, and galectin-3 **(I)**. Each point (filled circle) denotes relative protein expression which was normalized to β-actin and relative to a 0 DIV (acute slice) sample. Full-length blots: Supplementary Figures S4, S5. Statistical significance was tested by one-way ANOVA, and pairwise comparison was performed according to Tukey–Kramer multiple comparison test. NS; *p* > 0.05; *; *p* < 0.05; **; *p* < 0.01; ***; *p* < 0.001, (vs. 0 DIV). Data are presented as mean ± SD, based on four independent experiments (*n* = 4).

Previously, we reported that cultured rat hippocampal slices retained their neuronal activity for several weeks (Shinoda et al., 2005; Kamikubo et al., 2006). We recently showed that the reorganization and maturation of synapses occur in cultured hippocampal slices (Kamikubo et al., 2017). In the current study, we investigated the morphological features of neuronal and glial cells in hippocampal slice cultures after 3–62 days *in vitro* (DIV) (Supplementary Figure S1). We showed immunohistochemical staining images of 29 DIV in the middle of the culture period (Figures 2A–C). To observe glial cells, we performed immunostaining for GFAP and Iba1. GFAP is a hallmark intermediate filament protein in astrocytes (Hol and Pekny, 2015). Iba1 is a microglial macrophage-specific protein (Imai et al., 1996). In the slice culture, astroglia and microglia were distributed throughout the slice, and the arrangement of neurons was maintained. Hippocampal subregions, such as the dentate gyrus (DG), CA1, and CA3, could be clearly discerned (Figure 2A). APP and BACE1 expression in cultured hippocampal slices accumulated in the stratum radiatum, similar to the *in vivo* distribution (Kandalepas et al., 2013) (Supplementary Figure S2). Total protein levels in hippocampal slices did not change dramatically after 7 DIV (Figure 2D).

In a previous study, we showed that temporal changes in synaptic proteins in hippocampal slices indicate acute synaptic loss (0–4 DIV), followed by synapse formation (4–15 DIV) and maintenance (>15 DIV) phases (Muller et al., 1993; Kamikubo et al., 2017); therefore, in the current study, we used slices cultured at 0, 1, 4, 7, 14, 28, 42, and 56 DIV for further evaluation. We assessed the expression of markers for neurons, astrocytes, and microglia *via* immunoblotting analysis (Figures 2E–I). Expression levels of β-actin and βIII tubulin decreased immediately after cultivation and are stable after 4 DIV (Tukey’s Multiple Comparison Test, *p* > 0.05, versus 4 DIV). These results indicate that the number of neurons decreased immediately after plating the hippocampal slice on a filter membrane cup and stabilized after 7 days. However, the number of astrocytes and microglia increased 1 d after plating. The level of galectin-3, which is required for microglial activation in the injured brain (Lalancette-Hébert et al., 2012), was elevated for 1 week after preparation and then declined. PI assays showed that neural cells survived in 50 DIV cultures hippocampal slices (Supplementary Figure S3). These findings indicate that neural cells remained in the cultured hippocampal slices for over 8 weeks.

### Alternative cleavage of APP

**Figure 3:**
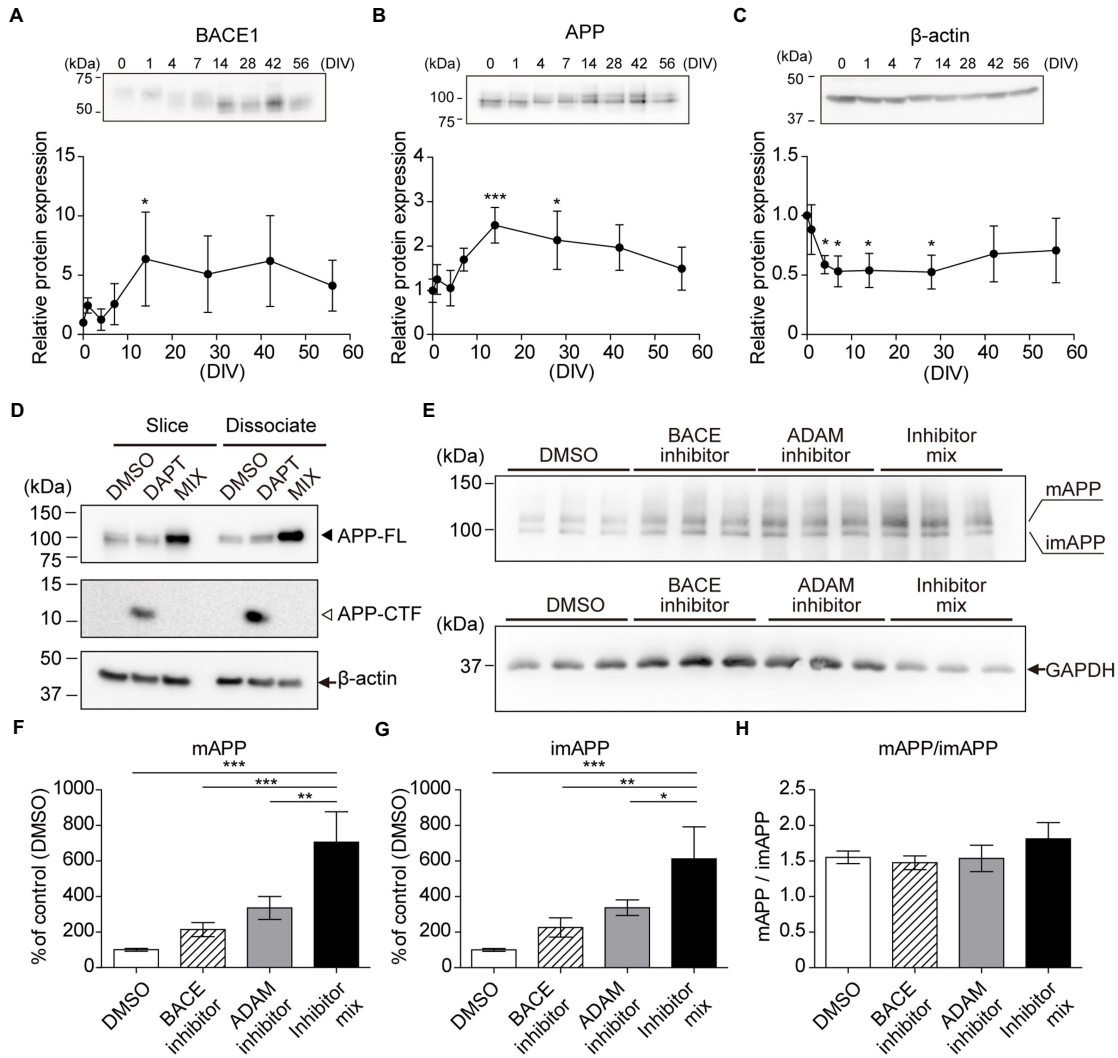
Chronological analysis of APP and BACE1 by immunoblotting. Immunoblotting analysis of hippocampal slices (0–56 days *in vitro*, DIV) using antibodies against BACE1 **(A)**, APP **(B)**, and GAPDH **(C)**. which was normalized to β-actin and relative to a 0 DIV sample. Each point (filled circle) denotes relative protein expression which was normalized to GAPDH and relative to a 0 DIV (acute slice) sample. Data were analyzed using one-way ANOVA, and pairwise comparison was performed according to Tukey–Kramer multiple comparison test. NS; *p* > 0.05, ^*^; *p* < 0.05, ^**^; *p* < 0.01, ^***^; *p* < 0.001 (vs. 0 DIV). Data are presented as mean ± SD, based on four independent experiments (*n* = 4). Full-length blots are shown in Supplementary Figure S5. **(D–H)** Immunoblotting analysis of APP cleavage. **(D)** Cultured hippocampal slices (slice, 14DIV) or dissociated hippocampal cells (dissociated, 14DIV) treated with γ-secretase inhibitor (DAPT, 10 μM) or secretase inhibitor mix (MIX; 10 μM DAPT, 50 μM ADAM inhibitor (GI254023X), and 2 μM BACE inhibitor (BACE inhibitor IV) for 24 h. β-actin served as a loading control. APP-FL: full-length APP. APP-CTF: APP C-terminal fragment. **(E–H)** Cultured hippocampal slices (14DIV, *n* = 3) treated with an ADAM inhibitor (GI254023X, 50 μM) and/or BACE1 inhibitor (BACE inhibitor IV, 2 μM) for 24 h. Mature APP-FL (mAPP) and immature APP-FL (imAPP) levels were evaluated quantitatively. Open bar, DMSO; stripe bar, BACE inhibitor; gray bar, ADAM inhibitor; black bar, BACE inhibitor and ADAM inhibitor mix. GAPDH served as a loading control. Full-length blots are shown in Supplementary Figure S6. Statistical significance was tested by one-way ANOVA, and pairwise comparison was performed according to Tukey–Kramer multiple comparison test. NS; *p* > 0.05; ^*^; *p* < 0.05; ^**^; *p* < 0.01; ^***^; *p* < 0.001. Data are presented as mean ± SD, based on three independent experiments (*n* = 3).

Previously, reorganization and maturation of neurons were reported in cultured hippocampal slices (Buchs et al., 1993; Muller et al., 1993). We evaluated the expression levels of BACE1, APP, and nicastrin using immunoblotting for several weeks. Our results indicated that their expression increased after 7 days (Figures 3A–C; Supplementary Figure S2). APP is cleaved by members of the α-or β-secretase family. α-Secretases are members of the ADAM family and are considered to be a part of the non-amyloidogenic pathway in APP processing (Selkoe, 1991; Hardy and Higgins, 1992). In accordance with previous reports (Stachel et al., 2004), DAPT treatment increased the level of APP carboxyl-terminal fragment (APP-CTF). Mixture of α-, β-, and γ-secretase inhibitors increased APP full-length (APP-FL) and abolished APP-CTF levels (Figure 3D). Furthermore, we cultured hippocampal slices (14DIV) with fresh SCM containing vehicle (DMSO) or each inhibitor after washing three times with 1 ml fresh SCM for 24 h. Treatment with each secretase inhibitor increased immature and mature APP-FL due to the reduction of APP cleavage. Treatment with both inhibitors blocked almost all the first steps of APP cleavage, further increasing the amount of APP-FL (Figures 3E–H).

### Biochemical analysis of Aβ production

**Figure 4:**
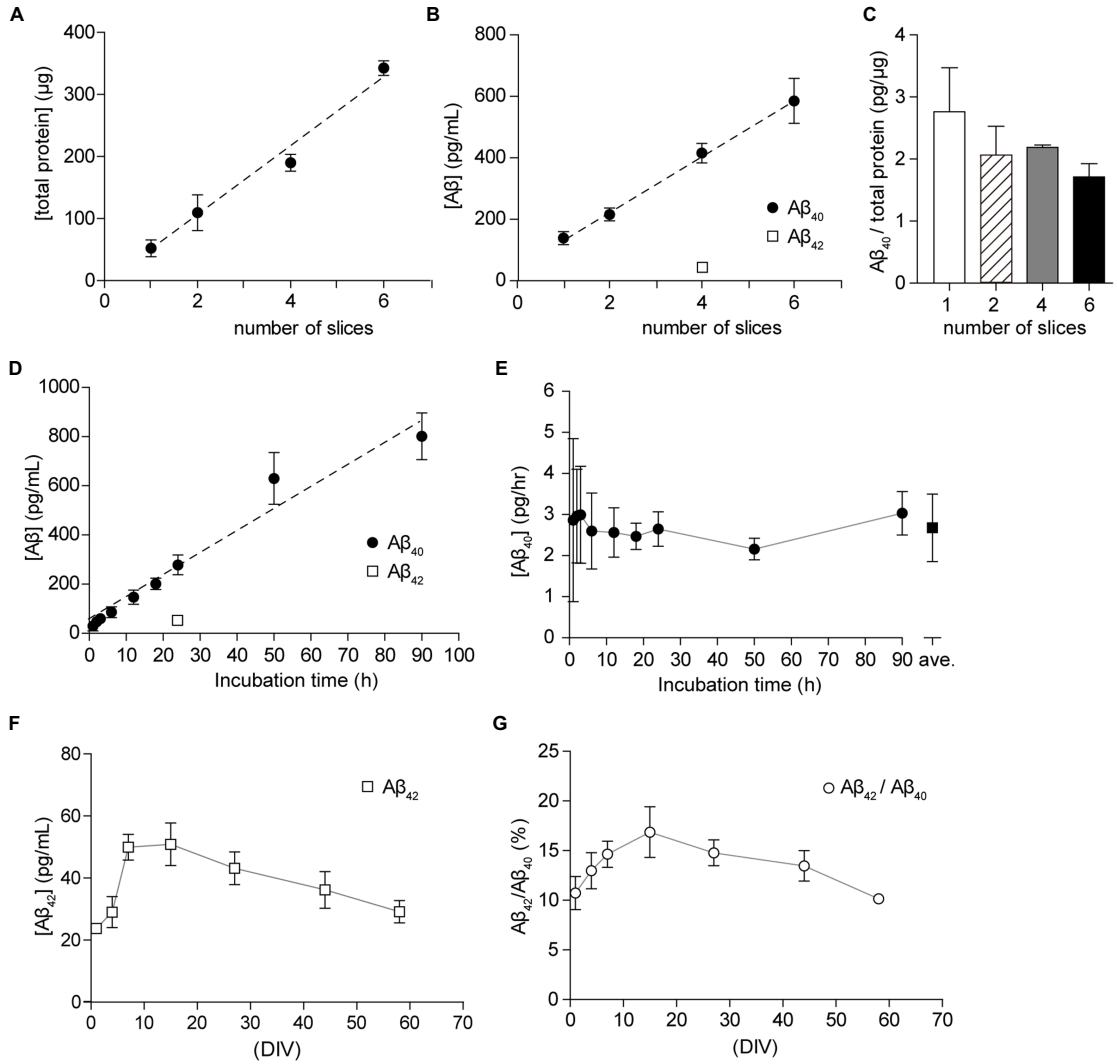
Culture condition and period dependency of the secretion of Aβ_40_. **(A)** Total protein in the lysate from 1, 2, 4, or 6 hippocampal slices (filled circles). Dashed line; linear regression. *R*^2^ = 0.9623, *p* < 0.0001. **(B)** Quantification of Aβ_40_ secreted from 1, 2, 4, or 6 cultured hippocampal slices (15 days *in vitro*, DIV) for 24 h (filled circles). Dashed line, linear regression of Aβ_40_. *R*^2^ = 0.9607, *p* < 0.0001. Quantification of Aβ_42_ secreted from 4 cultured hippocampal slices (15 days *in vitro*, DIV) for 24 h (open square). Results are presented as mean ± SD based on three independent experiments (*n* = 3). **(C)** The ratio of Aβ_40_ to total slice protein. Open bar, 1 slice; stripe bar, 2 slices; gray bar, 4 slices; black bar, 6 slices. Statistical significance was tested by one-way ANOVA, and pairwise comparison was performed according to Tukey–Kramer multiple comparison test. There was no significant difference between groups, *p* > 0.05. **(D)** Quantification of Aβ_40_ (filled circles) or Aβ_42_ (open square) secreted from a cultured hippocampal slice (15–21 DIV) for 1–90 h (Aβ_40_) or 24 h (Aβ_42_). Results are presented as mean ± SD based on four (1, 2, 3, 6, 12, 18 h) or eight (24, 50, 90 h) independent experiments (*n* = 4 or 8). Dashed line, linear regression of Aβ_40_. *R*^2^ = 0.9190, *p* < 0.0001. **(E)** Aβ_40_ secretion from a cultured hippocampal slice per 1 h. The amount of Aβ_40_ secretion is compensated for the residual medium (filled circle). Open square = an average of all dates (2.68 ± 0.82 pg/h, *n* = 48, 1–90 h). **(F,G)** Chronological quantitative analysis of the secretion of Aβ_42_ [**(F)**, open squares] or the ratio of Aβ_42_ to Aβ_40_ in SCM [**(G)**, open circles]. Four hippocampal slices were plated on a Millicell culture insert. For the Aβ assay, hippocampal slices were cultured with fresh SCM for 24 h at 37°C. Aβ_40_ and Aβ_42_ were quantified using ELISA.

Since organotypic cultures are artificial environments, we were able to control extracellular conditions, including temperature, oxygen content, nutritional factors, and neural activity. To elucidate the optimal conditions for the analysis of Aβ secretion, we examined the effect of the number of slices on the filter membrane cup and incubation period. Since reducing the number of slices in a cup allows for more efficient experiments, we plated 1 to 6 slices on a filter membrane cup for culture and incubated cultured slices with 1 ml fresh SCM for 24 h. We measured the total protein content of slices and Aβ in SCM from cultured hippocampal slices using a two-site ELISA assay (see Materials and Methods). The total protein content of the cultured hippocampal slice was 52.8 ± 9.3 μg. The correlation coefficient between Aβ_40_ and slice number was R^2^ = 0.9607 (*p* < 0.0001) (Figure 4A). In accordance with previous reports, a single hippocampal slice secreted 75–120 pg Aβ_40_ for 24 h (Figure 4B). Further analysis showed that the ratio of Aβ_40_ secretion to total protein amount was 1.7 to 2.8 pg/μg for 24 h (Figure 4C). There was no significant difference in Aβ_40_ secretion between groups (*p* > 0.05). To assess the incubation time for the Aβ_40_ assay, we cultured four hippocampal slices for 1–90 h with fresh SCM after washing three times with 1 ml fresh SCM. A statistically significant (*p* < 0.0001) strong correlation (R^2^ = 0.9190) was observed between Aβ_40_ secretion and the incubation time (Figure 4D). Our time-lapse analysis of Aβ secretion showed that a single hippocampal slice secreted 2.68 ± 0.82 pg for 1 h (Figure 4E).

### Chronological analysis of Aβ production

We investigated whether the amount of Aβ secreted changed depending on the culture period. To quantify Aβ secretion levels, we collected SCM-cultured hippocampal slices (1–58 DIV) for 24 h after washing three times with 1 ml fresh SCM. We previously reported that secretion of sAPPβ was between 3 and 6 ng/ml and secretion of Aβ_40_ was between 200 and 300 pg/ml during the monitoring period (Kamikubo et al., 2017). Aβ_40_ and Aβ_42_ are the most common isoforms of Aβ in the brain and are the main components of amyloid plaques found in AD brains. Here, we measured Aβ_42_ levels in the same SCM preparation using a two-site ELISA assay. The secretion of Aβ_42_ was 23 to 50 pg, and the ratio of Aβ_42_ to Aβ_40_ was 10 to 17% during the monitoring period (Figures 4F,G).

### Comparison of species differences in APP cleavage

**Figure 5:**
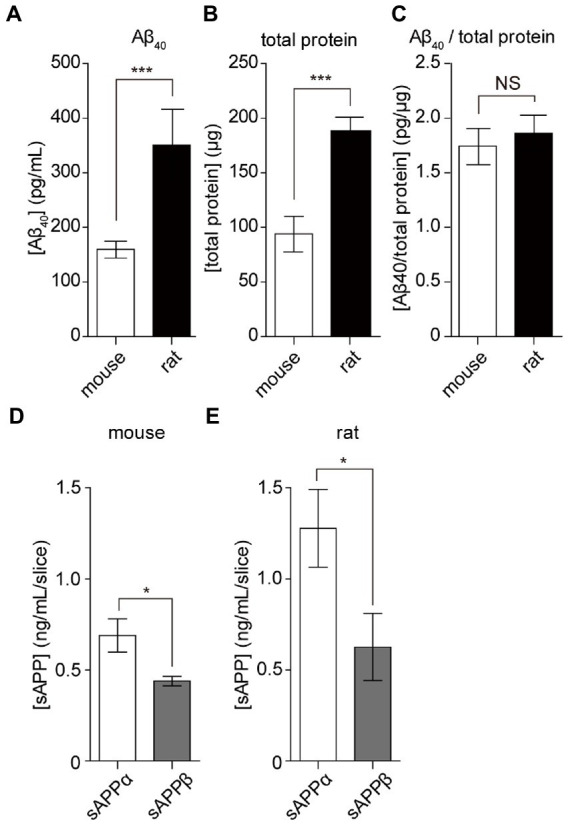
APP cleavage products from cultured mouse and rat hippocampal slices. Aβ_40_, sAPPα, and sAPPβ in SCM were quantified using ELISA. Four hippocampal slices were prepared from rats or mice plated on a filter membrane cup. **(A)** Quantification of Aβ_40_ secreted from hippocampal slices (15 days *in vitro*) cultured with fresh SCM for 24 h at 37°C. **(B)** Total protein levels in rat and mouse hippocampal slices. **(C)** Calculated data for the ratio of Aβ_40_ to total protein ratio. **(D,E)** Quantification of sAPPα and sAPPβ secreted from hippocampal slices cultured in fresh SCM for 24 h at 37°C. Statistical significance was determined using unpaired *t*-tests. NS; *p* > 0.05; *; *p* < 0.05; ***; *p* < 0.001. Data are presented as mean ± SD, based on three independent cultures (*n* = 3).

To apply hippocampal slice culture in various research fields, we assessed the amount of Aβ secretion by comparing rats and mice. The amount of Aβ_40_ secreted from cultured mouse hippocampal slices (15 DIV) was 160 ± 15 pg/ml for 24 h (Figure 5A). Although Aβ secreted from mouse hippocampus slices was less than that from rats (350 ± 65 pg/ml), there was no significant difference in Aβ secretion compared to the total protein content of slices (mice: 1.75 ± 0.3 pg/μg, rats: 1.86 ± 0.3 pg/μg, Figures 5B,C). APP is commonly cleaved by α-or β-secretase; both remove and release nearly the entire extracellular domain, referred to as sAPP (sAPPα and sAPPβ). This alternative cleavage is critical for elucidating the molecular mechanisms underlying AD pathology. We revealed that both sAPPα and sAPPβ are secreted from hippocampal slice cultures using the sAPP assay. The amount of secretion of sAPPα and sAPPβ from four cultured rat hippocampal slices was 1.30 ± 0.12 and 0.63 ± 0.11 ng, respectively. The amount of secretion of sAPPα and sAPPβ from four cultured mouse hippocampal slices was 0.69 ± 0.05 and 0.44 ± 0.01 ng, respectively (Figures 5D,E). These results indicated that α cleavage was dominant, similar to that in the model animal.

## Discussion

Herein, we present a method for preparing hippocampal slice cultures and consecutive analyses of APP processing. In this study, we sliced the hippocampus to a thickness of 400 μm. In many studies using brain organotypic cultures, the brain was sliced at 250–400 μm thick (Stoppini et al., 1991; Tominaga-Yoshino et al., 2002; Uesaka et al., 2012; Humpel, 2015). When thicker slices, such as 600 or 700 μm, are used, refluxing of the culture medium is required (Rambani et al., 2009), and fewer slices can be made from the hippocampus. Stable results were obtained by culturing four to five 400 μm thick slices in one cup and analyzing them together (Figure 4). Cultured hippocampal slices preserve the tissue architecture, neural circuits, and synaptic dynamics (Stoppini et al., 1991; Buchs et al., 1993; Muller et al., 1993). Because of individual differences and the difficulty of manipulation in animal models, comparative analyses before and after administration of the same preparation are not accessible. However, to evaluate drug efficacy, this comparative analysis before and after administration of the same culture preparation is meaningful. As primary dissociated neural cultures are vulnerable to drug washout during medium changes, a comparative study using the same preparation is extremely difficult. Filter membrane cups allow easy handling of preparations; therefore, we were able to collect and change the culture media without da mage and perform comparative analyses using the same preparations (Figure 4). Because the membrane cup is designed for disposable use, slice culture is costly. The use of self-made devices and filter membranes can reduce costs (Koyama et al., 2007).

Here, we showed the existence of microglia, astrocytes, and neurons in slices cultured for 2 months (Figure 2). Our chronological immunoblotting analysis indicated that glial cells proliferated for 1–2 weeks and were then maintained for several weeks (Figure 2G). In addition, the level of galectin-3, which is required for microglial activation and proliferation in the injured brain (Lalancette-Hébert et al., 2012), was elevated for a week after preparation and then declined (Figure 2I). The expression patterns of APP and BACE1 in cultured hippocampal slices accumulated at the stratum radiatum, similar to the *in vivo* distribution (Supplementary Figure S1). The expression levels of the synapse marker proteins synaptophysin and PSD95 decreased from 1 to 4 days and increased after 7 days (Kamikubo et al., 2017). Since synapses and neural circuits have formed and matured after 14DIV(Muller et al., 1993; Tominaga-Yoshino et al., 2002), quantitative analysis indicates that APP and BACE1 expression levels are stable (Tukey’s Multiple Comparison Test, *p* > 0.05, versus 14DIV, Figures 3A–C). Similar to previous studies (Kamikubo et al., 2017), immunoblotting analysis indicated that the electrophoretic mobility of BACE1 changed slightly after 4 DIV, which may represent the effect of the BACE1 maturation process. The newly synthesized BACE1 is cleaved at its prodomain before being transported to the trans-Golgi network (Benjannet et al., 2001). Further analysis of BACE1 intracellular localization is required; however, Aβ production is determined not only by BACE1 expression and enzyme activity but also by α-secretase and γ-secretase activity. Our results indicate that slices cultured for 2 or 3 weeks are suitable for analyzing neural functions and APP processing. We evaluated the differences between the slices and dissociated cultures; DAPT treatment accumulated APP-CTF to the same extent in both dissociated and slice cultures (Figure 3D). The α-secretase family of proteins alternatively cleaves APP within the Aβ sequence. Thus, α-cleavage precludes Aβ formation and is considered part of the non-amyloidogenic pathway in APP processing (Lammich et al., 1999). In cultured hippocampal slices, alternative cleavage of APP is conserved (Figures 3F–H); thus, the application of the α-secretase inhibitor increases the secretion of Aβ (Kamikubo et al., 2017). On the other hand, the ratio of mature APP-FL (mAPP) to immature APP-FL (imAPP) was not altered by secretase inhibitor treatment (Figure 3H). This result indicates that secretase activity does not affect APP maturation. Secretase inhibition may result in accumulation of only mAPP or both mAPP and imAPP. Further investigation is needed to explain why secretase inhibition increases both mature and immature forms of APP. Since secretases are required for neuronal growth and maturation (Chow et al., 2010), inhibition of both secretases could have adversely affected neurons and reduced GAPDH expression (Figure 3E). Western blotting is at best a semi-quantitative method and dependent upon the proper choice of loading controls. Long-term incubation may alter the expression levels of β-actin and GAPDH, which are generally considered loading controls. Therefore, further study is needed to compare different culture periods. In this report, immunostaining of slice cultures was performed to confirm the survival of neurons and glial cells and their distribution over a 2-month period.

Aβ is formed after the sequential cleavage of APP by β-and γ-secretase, which accumulates in the central nervous system and subsequently initiates neural dysfunction. Excessive accumulation of Aβ results in aggregation and amyloid deposition in the brains of AD patients (Blennow et al., 2006). γ-Secretase cleaves within the transmembrane region of APP and can generate several isoforms of 30–51 amino acid residues in length (Olsson et al., 2014). Aβ_40_ and Aβ_42_ are the most common isoforms of Aβ in the brain and are the main components of amyloid plaques found in AD brains. Since the pathological changes of AD progress slowly, a model for continuously analyzing central nervous function for an extended period is required. This study evaluated a method to continuously investigate Aβ formation using cultured hippocampal slices. To estimate Aβ production, we measured Aβ_40_ and Aβ_42_ secretion from hippocampal slices using a two-site ELISA assay. In the culture medium, four hippocampal slices secreted 200–400 pg Aβ_40_ and 23–50 pg Aβ_42_ for 24 h (Kamikubo et al., 2017). These data indicated that 2.0–4.0 pg Aβ_40_ and 0.2–0.5 pg Aβ_42_ were secreted by a cultured hippocampal slice in 1 h. According to previous reports (Kamenetz et al., 2003), the ratio of Aβ_42_ to Aβ_40_ is between 10 and 17% (Figure 4). Neurodegenerative diseases are well characterized in primary cultures of rats or mice. However, differences between species are rarely analyzed. We, therefore, evaluated APP processing in hippocampal slice cultures from these rodents. Examination of the differences between species revealed no difference between the amount of Aβ_40_ and sAPP secretion in mouse and rat hippocampal slices (Figure 5). Cultured rodent hippocampal slices are useful for analyzing APP processing. The Aβ_40_ form is the most common of the two; however, human Aβ_42_ is more fibrillogenic and is thus associated with disease states (Yin et al., 2007). Since rodent Aβ is poorly aggregated (Jankowsky et al., 2007), cultured hippocampal slices are not a good platform for analyzing Aβ aggregation and accumulation. The addition of chemically synthesized or recombinant human Aβ should be used to analyze the regulation of endogenous Aβ production by using antibodies specific to mouse/rat Aβ.

Furthermore, we attempted to optimize the incubation period and number of slices for the analysis of Aβ. We placed 1 to 6 hippocampal slices on a membrane cup for 1 week of culture and incubated these slices for 24 h with fresh SCM for the Aβ assay. Aβ concentration increased linearly with the number of cultured slices (Figure 4). For time-lapse analysis of Aβ secretion, we cultured four hippocampal slices and incubated them for 1–90 h with fresh SCM after washing three times with 1 ml fresh SCM. Our results indicate that a single hippocampal slice secreted 2.68 ± 0.82 pg for 1 h (Figures 4B,C). The dispersion of the Aβ assay indicated that the carryover medium significantly affected the measurement results with short-term incubation. When many slices are placed on a membrane cup, it is possible to analyze Aβ production, but proper placement of slices is difficult. We suggest that it is appropriate to incubate 2 to 6 hippocampal slices in a membrane cup.

Slice cultures are usually derived from early postnatal or embryonic rodents. Because an essential cytoarchitecture has already been established, neural circuits are still immature, and early postnatal periods (days 0 to 10) are ideally suited for culturing. Some attempts have been made to culture adult tissues of animal models to elucidate age-related neurodegenerative diseases (Mewes et al., 2012). Slice cultures have been used in various research fields, including physiology, pharmacology, endocrinology, biochemistry, and development of pathology (Ikegaya, 1999; Humpel, 2015; Hiragi et al., 2017; Richter et al., 2020). Since rodent Aβ has low aggregation properties, it is possible to analyze Aβ aggregation and toxicity by exogenous administration of human Aβ, which has high aggregation properties. Other groups have been able to reproduce pathological protein aggregates deposition by injection seed of Aβ aggregates, α-synuclein aggregates, or extracts from the brains of patients with neurodegenerative diseases (Wu et al., 2020). We cultured hippocampal slices for several months using the methods described in previous reports (Kamikubo et al., 2006, 2017). Using slice cultures of hippocampi derived from early-onset models of neurodegenerative diseases (Saito et al., 2014; Novotny et al., 2016; Scheiblich et al., 2021), we can reveal the early processes of these diseases. The use of brain slice cultures of wild-type rodents to construct a model of neurodegenerative diseases may avoid the compensatory mechanism of genetic modification. Furthermore, by using cultured brain slices, we can analyze embryonically lethal genetic modifications. Specific cells can be removed from cultured hippocampal slices with drug administration (Vinet et al., 2012; Richter et al., 2020). Co-culturing slices from the brains of mice with distinct genetic modifications enables analyses that are not feasible *in vivo* (Uesaka et al., 2012, 2018). Brain organ culture only applies to analyzing a limited number of brain regions, making diagnosing neurodegenerative diseases in a wide range of brain regions challenging. We anticipate that hippocampal slice culture can be used as an *ex vivo* model to reveal the molecular and cellular basis of neuropsychiatric disorders.

This study provides a method for consecutive analysis of protein secretion from brain slices to study neurodegenerative diseases. Hippocampal slice culture is an experimental system that effectively maintains the *in vivo* neuronal network and is a convenient method to study the cellular and molecular mechanisms underlying neuropsychiatric disorders, including AD, and to evaluate therapeutic approaches for such diseases.

## Data availability statement

The original contributions presented in the study are included in the article/Supplementary material, further inquiries can be directed to the corresponding author.

## Ethics statement

The animal study was reviewed and approved by Animal Experimentation Committee of Juntendo University.

## Author contributions

YK conceived and designed the study and wrote and reviewed the manuscript. YK, YZ, HJ, KN, YH, and NT contributed to investigation and data analysis. YK and TS supervised the study. All the authors approved the final version of the manuscript.

## Funding

This work was supported by JSPS KAKENHI Grant Numbers JP20K07765 and JP17K09040 (to YK) and grants-in-aid from the TERUMO LIFE SCIENCE FOUNDATION (to YK).

## Conflict of interest

The authors declare that the research was conducted in the absence of any commercial or financial relationships that could be construed as a potential conflict of interest.

## Publisher’s note

All claims expressed in this article are solely those of the authors and do not necessarily represent those of their affiliated organizations, or those of the publisher, the editors and the reviewers. Any product that may be evaluated in this article, or claim that may be made by its manufacturer, is not guaranteed or endorsed by the publisher.

## Abbreviations

AD, Alzheimer’s disease
APP, amyloid precursor protein
Aβ, amyloid β peptide
DG, dentate gyrus
DIV, days *in vitro*
GBSS, Gay’s BSS
PBS, phosphate-buffered saline
OHP, overhead projector
TBS, Tris-buffered saline
