# Supplementary figures and images for: Ex vivo analysis platforms for monitoring amyloid precursor protein cleavage
Source: Front Mol Neurosci. 2023 Jan 6;15:1068990. doi: 10.3389/fnmol.2022.1068990 (PMC9852844; doi:10.3389/fnmol.2022.1068990)

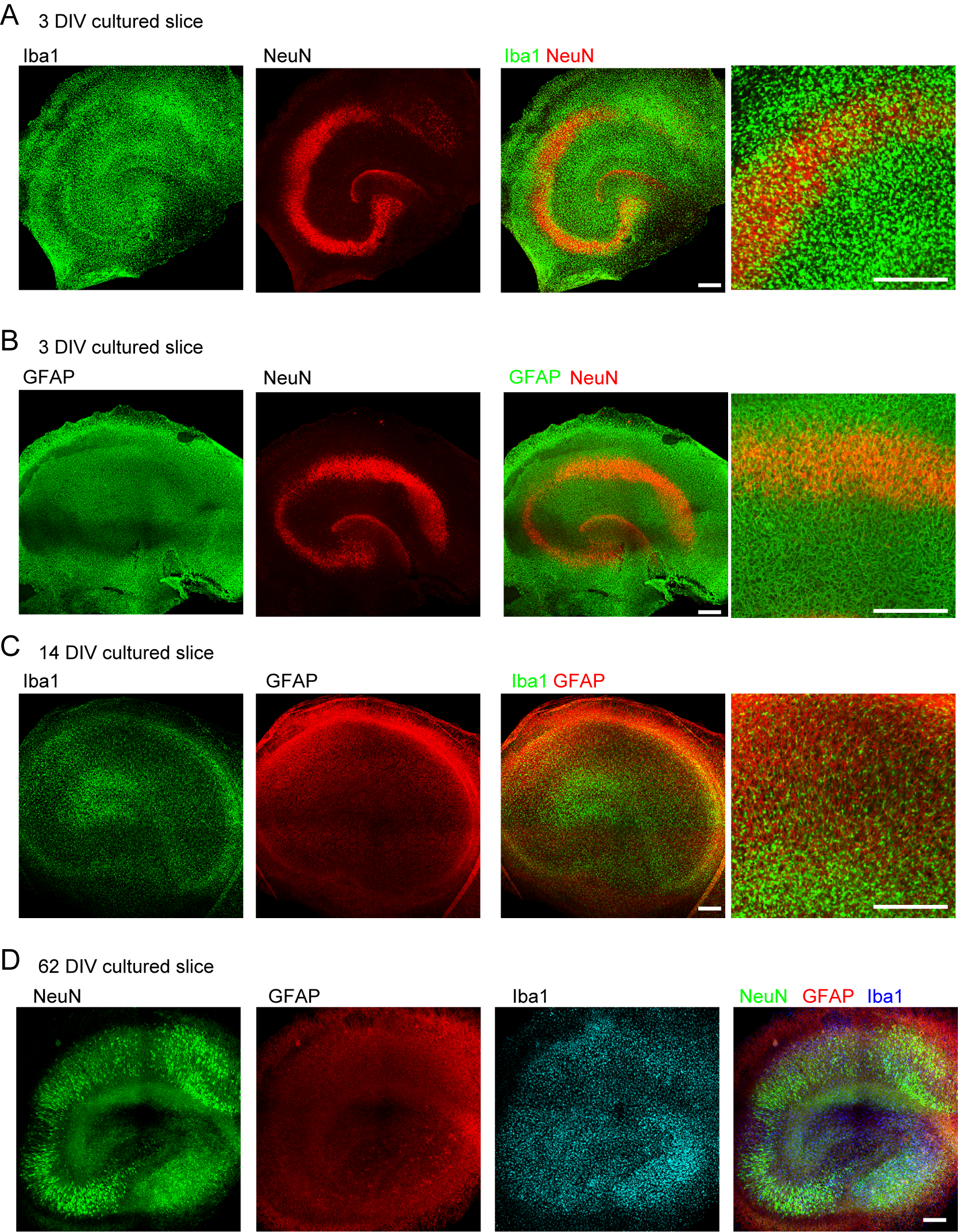

Supplement: Supplementary file 1 [file Image_1.TIF]

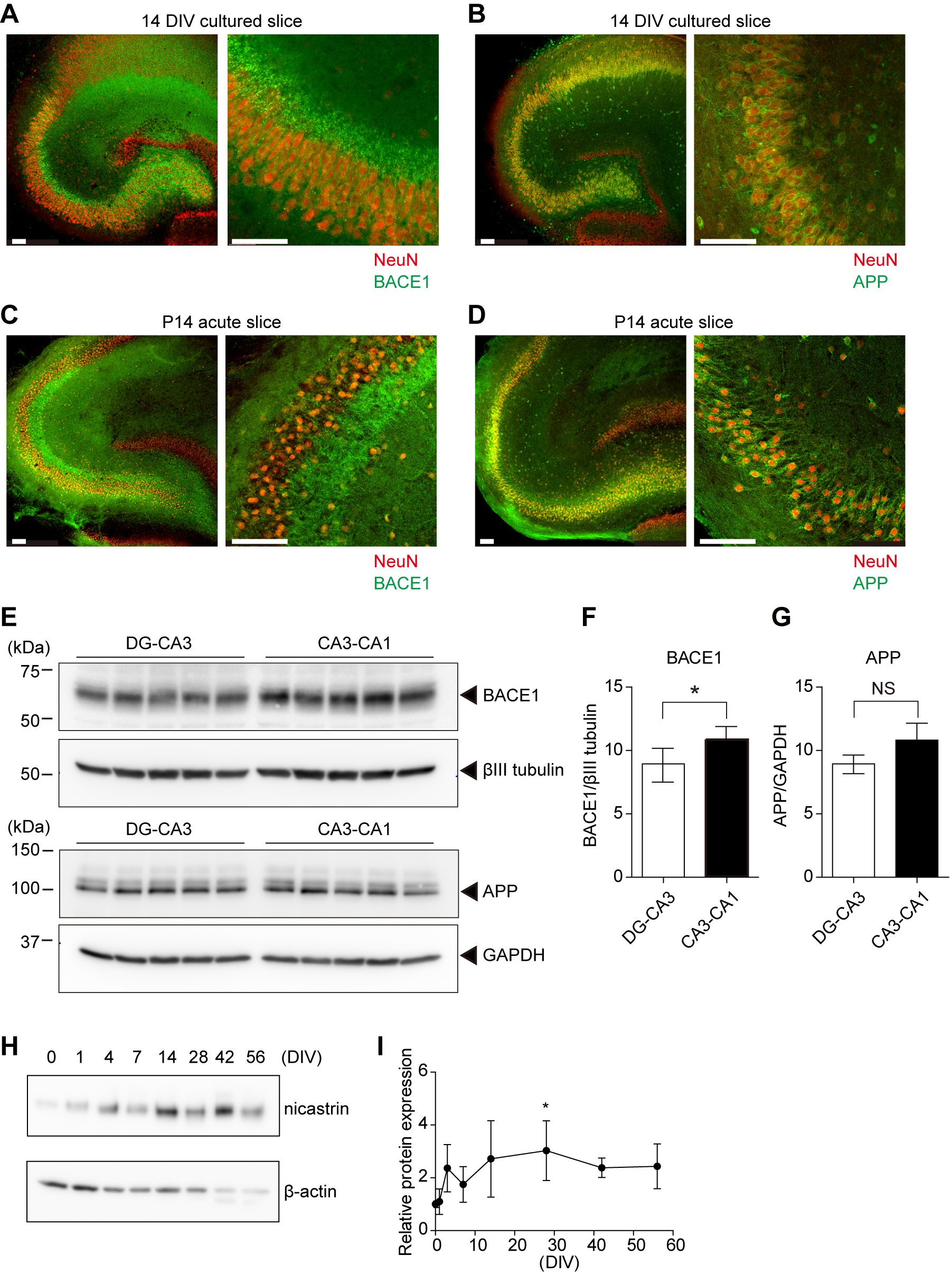

Supplement: Supplementary file 2 [file Image_2.TIF]

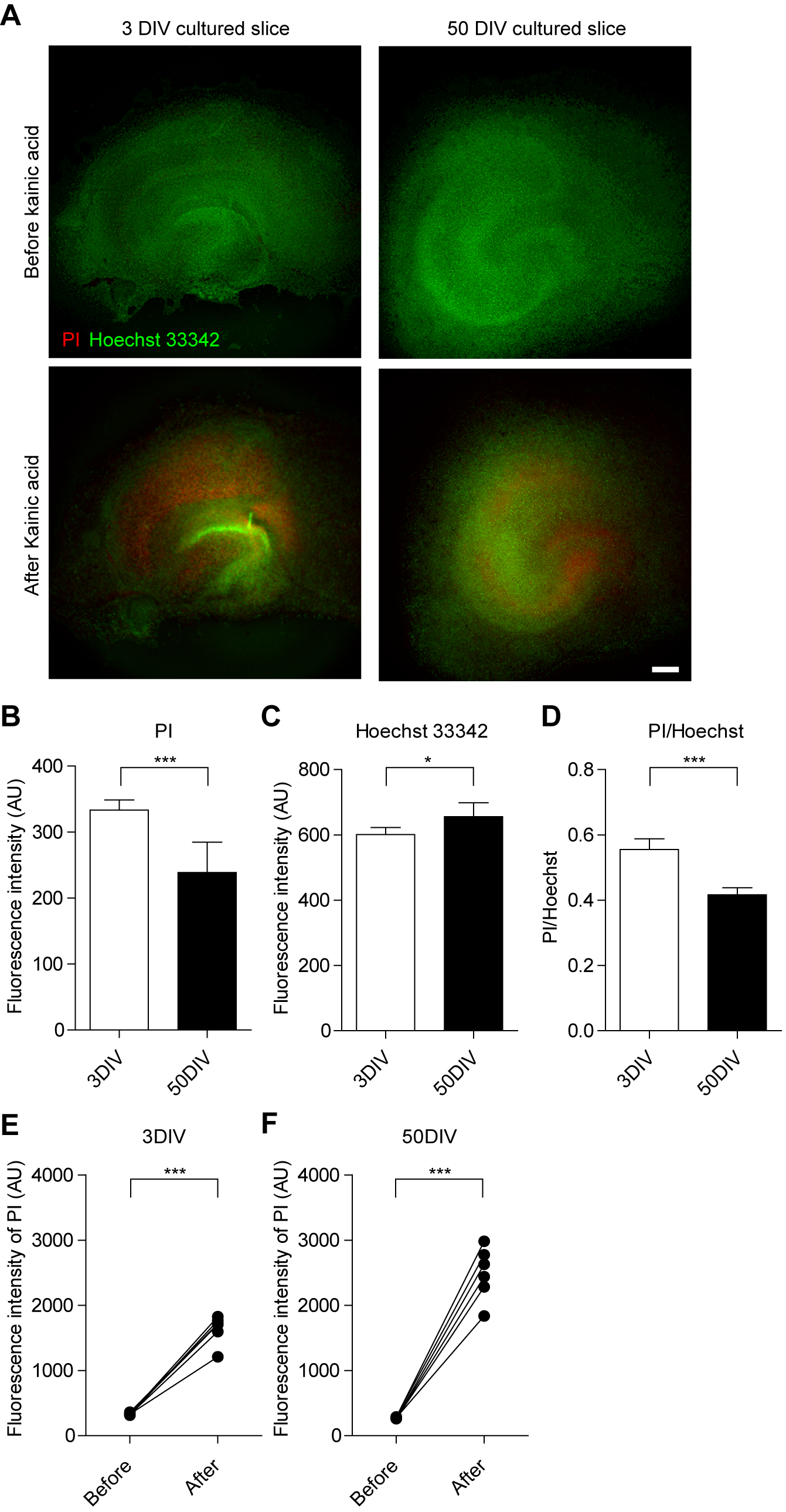

Supplement: Supplementary file 3 [file Image_3.TIF]

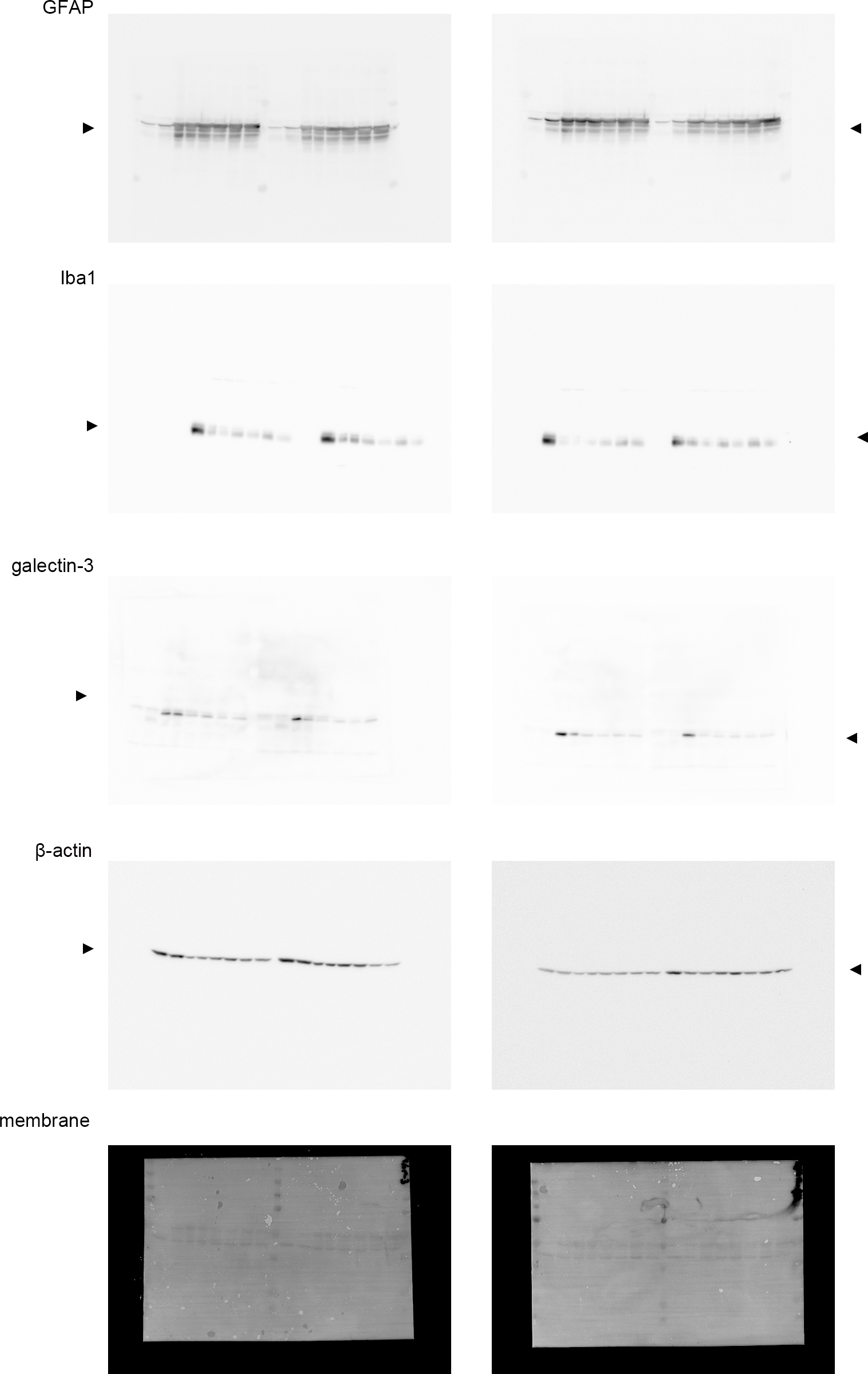

Supplement: Supplementary file 4 [file Image_4.TIF]

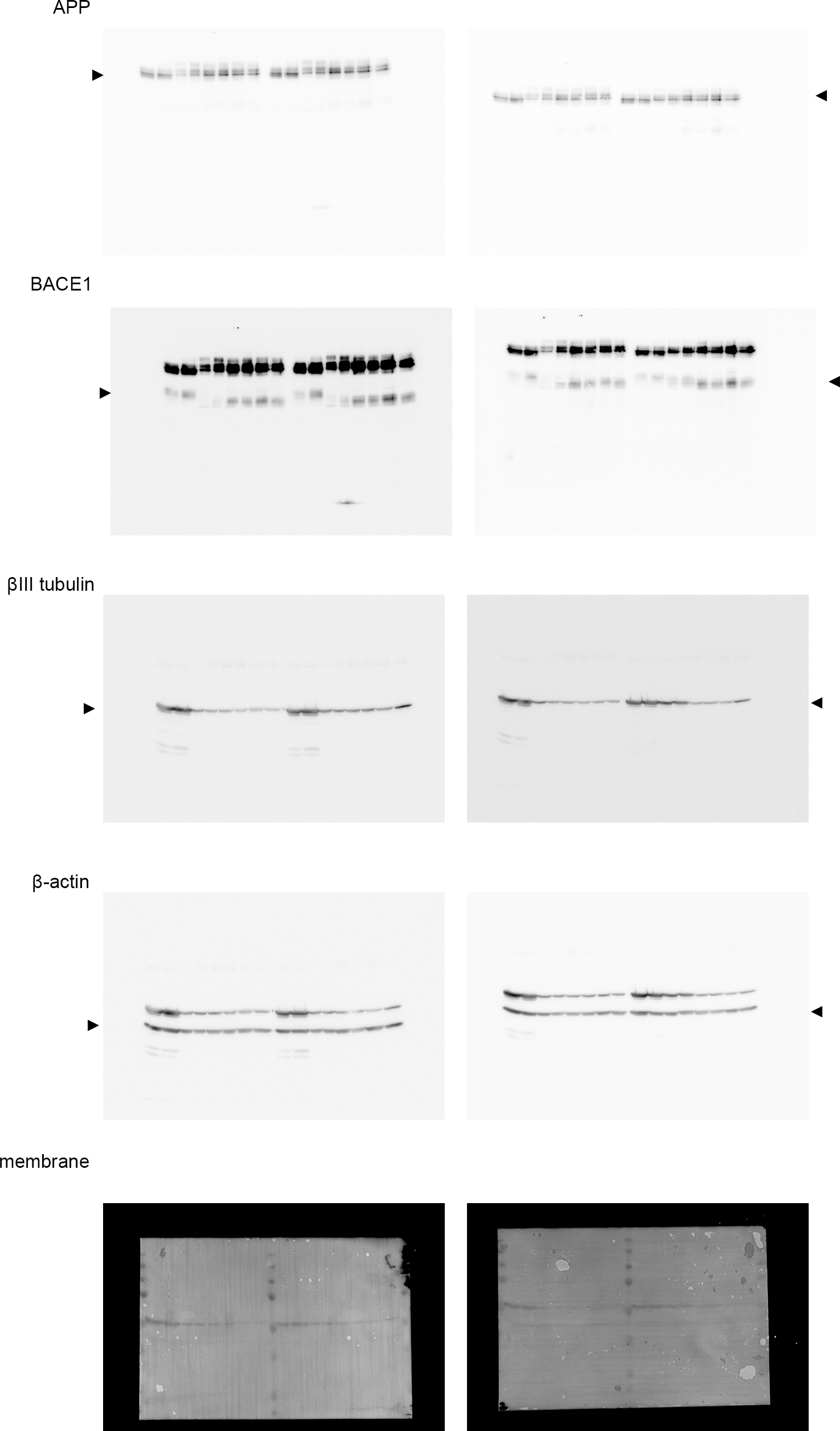

Supplement: Supplementary file 5 [file Image_5.TIF]

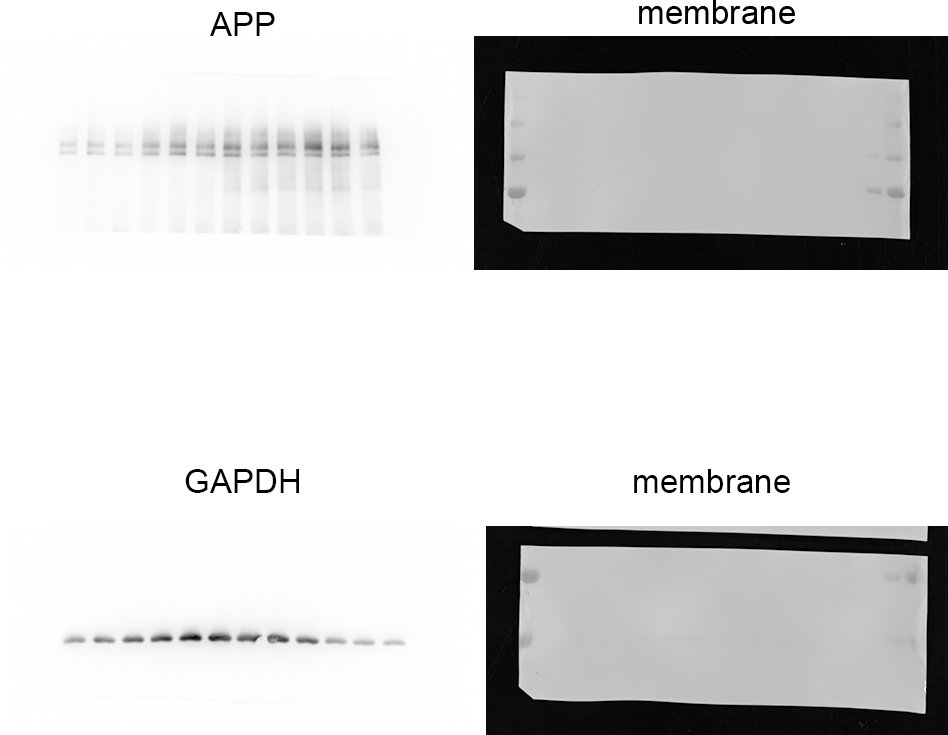

Supplement: Supplementary file 6 [file Image_6.TIF]

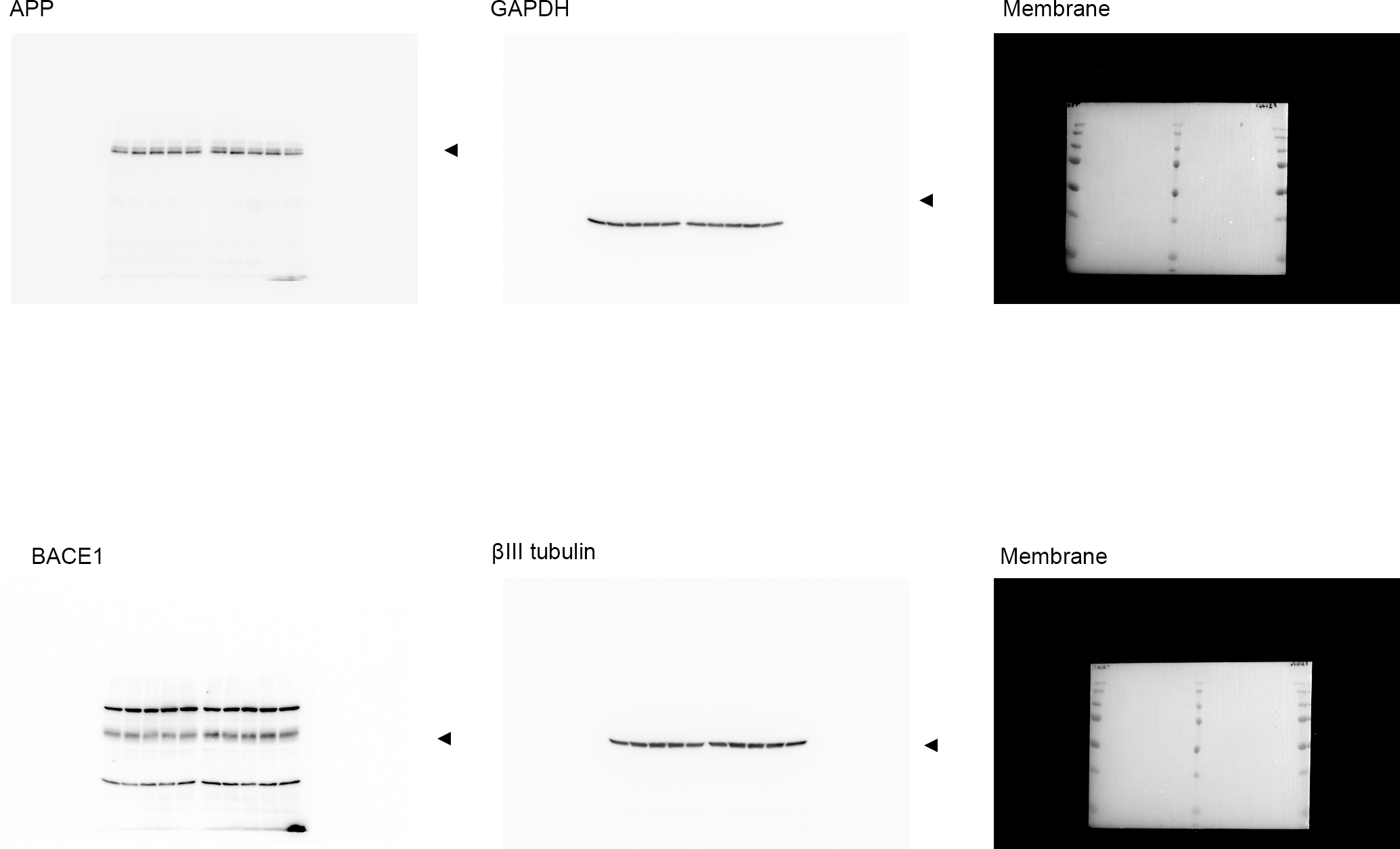

Supplement: Supplementary file 7 [file Image_7.TIF]

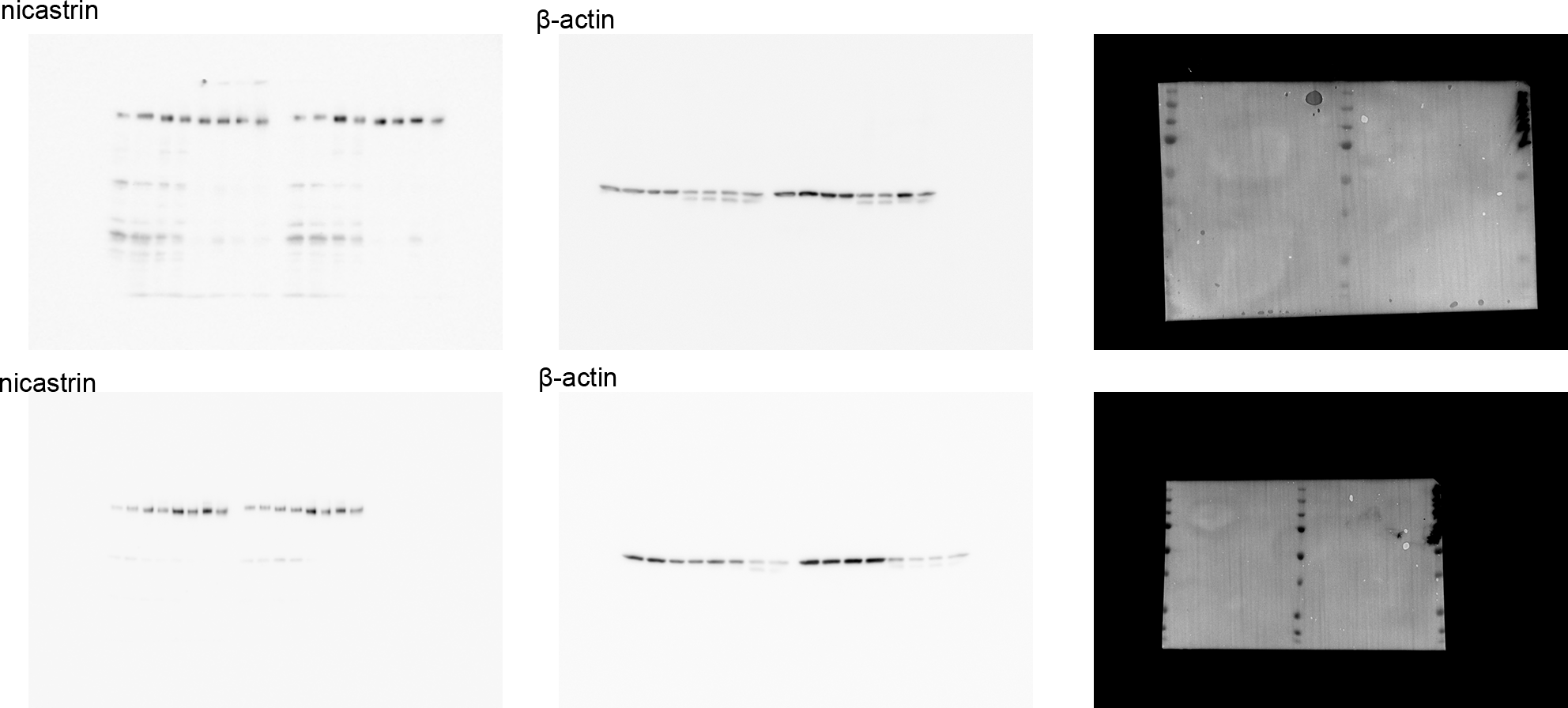

Supplement: Supplementary file 8 [file Image_8.TIF]
